# Machine learning for diabetic foot care: accuracy trends and emerging directions in healthcare AI

**DOI:** 10.3389/fpubh.2025.1613946

**Published:** 2025-07-18

**Authors:** Pei-Chun Lin, Tsai-Chung Li, Tzu-Hsuan Huang, Ying-Lin Hsu, Wen-Chao Ho, Jia-Lang Xu, Ching-Liang Hsieh, Zih-En Jhang

**Affiliations:** ^1^Department of Public Health, College of Public Health, China Medical University, Taichung, Taiwan; ^2^Institute of Population Health Sciences, National Health Research Institutes, Miaoli, Taiwan; ^3^Department of Audiology and Speech-Language Pathology, College of Medical and Health Sciences, Asia University, Taichung, Taiwan; ^4^Doctoral Program in Big Data Analytics for Industrial Applications, Nation Chung Hsing University, Taichung, Taiwan; ^5^Department of Applied Mathematics and Institute of Statistics, National Chung Hsing University, Taichung, Taiwan; ^6^Department of Computer Science and Information Engineering, Chaoyang University of Technology, Taichung, Taiwan; ^7^Department of Chinese Medicine, China Medical University Hospital, Taichung, Taiwan; ^8^Graduate Institute of Acupuncture Science, College of Chinese Medicine, China Medical University, Taichung, Taiwan; ^9^Chinese Medicine Research Center, China Medical University, Taichung, Taiwan; ^10^Department of Medical Imaging, Changhua Christian Hospital, Changhua, Taiwan

**Keywords:** diabetic foot, machine learning, thermal imaging, clinical data analysis, internet of things, artificial intelligence in healthcare

## Abstract

**Background:**

Diabetic foot is a common and debilitating complication of diabetes that significantly impacts patients’ quality of life and frequently leads to amputation. In parallel, artificial intelligence (AI), particularly machine learning (ML), has emerged as a powerful tool in healthcare, offering novel solutions for disease prediction, monitoring, and management. Despite growing interest, a systematic overview of machine learning applications in diabetic foot research is still lacking.

**Objective:**

This study aims to systematically analyze recent literature to identify key trends, focus areas, and methodological approaches in the application of machine learning to diabetic foot research.

**Data sources:**

A comprehensive literature search was conducted across three major databases: Web of Science (WoS), IEEE Xplore, and PubMed. The search targeted peer-reviewed journal articles published between 2020 and 2024 that focused on the intersection of machine learning and diabetic foot management.

**Eligibility criteria and study selection:**

Articles were included if they were indexed in the Science Citation Index (SCI) or Social Sciences Citation Index (SSCI), published in English. They explored the use of machine learning in diabetic foot-related applications. After removing duplicates and irrelevant entries, 25 original research articles were included for review.

**Results:**

There has been a steady increase in publications related to machine learning in diabetic foot research over the past 5 years. Among the 25 studies included, image analysis was the most prevalent theme (12 articles), dominated by thermal imaging applications (10 articles). General clinical imaging was less common (2 articles). Seven studies focused on structured clinical data analysis, while six explored IoT-based approaches such as smart insoles with integrated sensors for real-time foot monitoring. Citation analysis showed that Computers in Biology and Medicine and Sensors had the highest average citation rates among journals publishing multiple relevant studies.

**Conclusion:**

The integration of machine learning into diabetic foot research is rapidly evolving; it is characterized by growing diversity in data modalities and analytical techniques. Thermal imaging remains a key area of interest, while IoT innovations show promise for clinical translation. Future studies should aim to incorporate deep learning, genomic data, and large language models to further enhance the scope and clinical utility of diabetic foot research.

## Introduction

Diabetes has become one of the top 10 causes of death worldwide, and the rising mortality rate in certain regions indicates suboptimal diabetes management (1). In the United States, the prevalence of diabetes increased from 10.2% in 2012 to 12.1% in 2022, contributing significantly to both health and economic burdens (2). In Taiwan, the diabetic population grew by 66% between 2005 and 2014 (3). These data collectively highlight that diabetes is a serious and non-negligible disease. Diabetes is primarily classified into two types: type 1 and type 2. Type 1 diabetes is characterized by the loss of pancreatic β-cells, leading to insulin deficiency and subsequent hyperglycemia (4). Type 2 diabetes, a more common form, is often associated with other metabolic abnormalities such as hypertension and dyslipidemia (5). It is mainly characterized by defects in insulin secretion and insulin resistance, and accounts for approximately 90–95% of all diabetes cases (6).

With the rise of smart healthcare, numerous researchers have published studies aiming to support the medical field and alleviate the burden on healthcare providers. The applications of artificial intelligence (AI) and machine learning (ML) in healthcare are increasingly diverse. Machine learning is a way for computers to automatically learn from data and obtain rules and use the rules to make model predictions. There are several different learning paradigms for machine learning: supervised learning, unsupervised learning, semi-supervised learning, and reinforcement learning. In nursing care, machine learning methods have been employed to reduce the risk of patient falls (7, 8). In critical care, researchers have utilized machine learning and deep learning techniques to monitor patients and predict the appropriate timing for ventilator weaning (9, 10). In stroke management, AI has been applied to predict patient recovery outcomes (11), while in lung cancer, deep learning approaches have been used for early diagnosis and prognosis prediction (12). These advancements clearly demonstrate the significant progress and potential of smart healthcare. Among diabetic complications, diabetic retinopathy is one of the most common, and the integration of AI with telemedicine has the potential to greatly improve diabetic retinopathy screening and management (13). Similarly, in the context of diabetic foot, the early application of AI technologies could prevent delayed diagnoses, thereby effectively reducing the incidence and severity of this complication (14).

This study focuses on a literature review to investigate the current applications of machine learning in diabetic foot research. The identified studies are categorized into three major areas: (1) clinical imaging-based research on diabetic foot, (2) clinical numerical data-based research, and (3) research on the application of Internet of Things (IoT) technologies.

## Methods

### Information sources

A comprehensive literature search was conducted to identify relevant studies on the application of clinical numerical data and medical imaging in the management of diabetic foot. Three major academic databases were utilized: Web of Science (WOS), IEEE Xplore, and PubMed. Web of Science was selected for its extensive coverage of multidisciplinary peer-reviewed literature, including journals indexed in the Science Citation Index (SCI) and Social Sciences Citation Index (SSCI). IEEE Xplore was included to capture research within the domains of engineering and computational sciences, both of which are highly relevant to machine learning applications. PubMed, a leading biomedical database, provided access to clinically focused studies on diabetic foot-related complications. The search was limited to peer-reviewed articles published between 2019 and 2024 to ensure the inclusion of up-to-date research. A keyword-based strategy was employed, using the terms “Diabetic Foot” and “Machine Learning” in the abstract fields.

### Selection of sources of evidence

**Figure 1:**
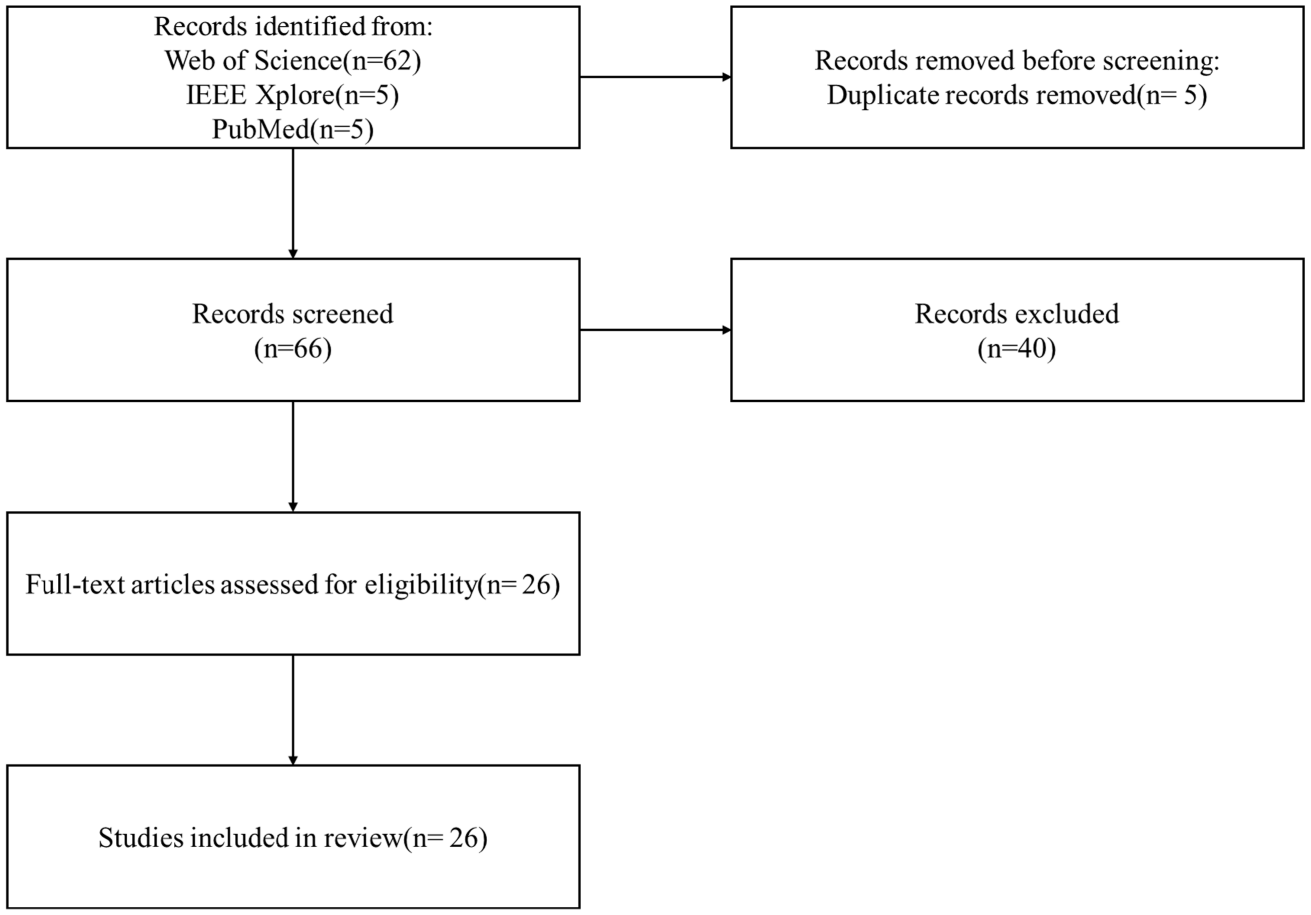
PRISMA flow diagram of study selection.

The initial search identified 72 relevant articles. After removing duplicate entries across the databases, 66 unique articles were retained for eligibility screening. A multi-phase screening process was subsequently conducted; it included title and abstract screening, followed by full-text evaluation based on predefined inclusion and exclusion criteria. Studies were selected based on their methodological rigor, originality, and relevance to the research focus. Articles that were not aligned with the study objectives or that constituted systematic reviews were excluded. In total, 40 articles were excluded during the screening process, resulting in the inclusion of 26 original research articles for in-depth qualitative and quantitative analysis. The study selection process is depicted in the PRISMA flow diagram shown in Figure 1, while the specific inclusion and exclusion criteria are summarized in Table 1.

**Table 1:** Classification of included and excluded articles.

Included/excluded	Clean rules	Count
Included	Clinical imaging-based research on diabetic foot Clinical data-based research on diabetic foot Internet of Things (IoT) applications research on diabetic foot	26
Excluded	Genetics and molecular biology research on diabetic foot Studies not meeting inclusion criteria Systematic review articles Studies without application of machine learning techniques	40

## Results

### Results of individual sources of evidence

#### Clinical imaging-based research on diabetic foot

Thermal imaging is a technique that captures infrared radiation emitted by objects to generate visual images. Thermal imaging devices detect this radiation and convert it into visual representations. Castillo-Morquecho et al. (15) utilized thermal imaging to measure foot temperature and applied principal component analysis (PCA) along with support vector machines (SVM) for classification, achieving an accuracy of 90%. Their study also found a positive correlation between average foot temperature, glycated hemoglobin (HbA1c), and body mass index (BMI) (15). Khandakar et al. (16) employed thermal imaging to predict variations in the Thermal Change Index (TCI). They applied machine learning techniques for feature extraction and image classification of TCI, concluding that multilayer perceptron models achieved an accuracy of 90.1% (16). Khandakar et al. (16) used a diabetic thermogram dataset and applied k-means clustering to categorize the severity of foot ulcers. They compared the performance of machine learning models and convolutional neural networks (CNNs), finding that both approaches provided satisfactory predictive results (17). Khosa et al. (18) applied SVM-based machine learning methods to extract features from thermal images for diabetic foot classification (18). Khandakar et al. (19) further used machine learning techniques for feature scoring, selection, and optimization, by comparing traditional CNN models. They reported that the AdaBoost model achieved a 97% F1-score using only 10 features (19). Evangeline et al. (20) used the random forest algorithm to classify patients with and without Diabetic Foot Syndrome (DFS), demonstrating that applying the Synthetic Minority Oversampling Technique (SMOTE) improved model accuracy (20). Cruz-Vega et al. (21) compared machine learning and deep learning algorithms for classifying diabetic foot thermal images and found that traditional artificial neural networks (ANN) and SVM models achieved better predictive performance after feature extraction (21). Hernandez-Guedes et al. (22) utilized Lasso, random forest, concrete dropout, and variational dropout methods for feature extraction, followed by SVM classification; they achieved promising F1-scores of approximately 90% (22). Arteaga-Marrero et al. (23) compared features across different datasets, including the STANDUP and INAOE datasets, and identified the lateral plantar artery (LPA) and medial calcaneal artery (MCA) as the most relevant features (23). Alshayeji et al. (24) developed a machine learning model for real-time diagnosis of diabetic foot ulcers; they employed a combination of Scale-Invariant Feature Transform (SIFT), Speeded Up Robust Features (SURF), and the Bag of Features (BOF) techniques for feature extraction, achieving an accuracy of 97.81% (24). Almufadi and Alhasson (25) compared various CNN models to classify diabetic foot ulcers with or without ischemia or infection; they found that incorporating the AdaBoost algorithm into EfficientNetB0 yielded better predictive performance compared to using EfficientNetB0 alone (25). Alzubaidi et al. (26) proposed a novel algorithm, DFU_QUTNet, for distinguishing between normal and abnormal skin in diabetic foot images. Their model utilized neural networks for feature extraction and SVM for classification, achieving high predictive accuracy (26). Wang et al. (27) proposed a wound boundary determination method based on the Associative Hierarchical Random Field (AHRF) framework, which demonstrates strong robustness against variations in lighting and camera angle, achieving superior overall accuracy in boundary recognition performance (27).

#### Clinical data-based research on diabetic foot

Hong et al. (28) utilized high-risk features from older adult diabetic patients to assess the risk of diabetic foot ulcer (DFU) recurrence. They trained a support vector machine (SVM) model, and achieved an accuracy of 93% (28). Nanda et al. (29) employed biochemical examination indicators to classify the presence or absence of DFUs and further categorized the types of ulcers. Their study identified interleukin-10 (IL-10) and uric acid as key markers for differentiating ulcer severity (29). Oei et al. (30) developed a risk prediction model for lower extremity amputation in patients with diabetic foot ulcers. They trained the model using 51 clinical features and applied SHapley Additive exPlanations (SHAP) for model interpretation, identifying total white cell (TWC) count, comorbidity score, and red blood cell count as the most significant contributors, while total white cell count, eosinophils, and necrotic eschar were found to have the greatest impact (30). Wang et al. (31) used nine variables: random blood glucose, years with diabetes, cardiovascular diseases, peripheral arterial diseases, DFU history, smoking history, albumin, creatinine, and C-reactive protein, to predict the risk of amputation in stage III DFU patients. Their results indicated that the XGBoost algorithm achieved the best predictive performance (31). Matsinhe et al. (32) applied machine learning models to predict mortality among patients with diabetic foot sepsis (DFS), highlighting that during the COVID-19 pandemic, major amputation and mortality rates significantly increased. The random forest model was found to effectively predict the risks of both major amputation and death (32). Haque et al. (33) noted that diabetic sensorimotor polyneuropathy (DSPN) is a serious long-term complication of diabetes. They utilized machine learning models with factors from the Michigan Neuropathy Screening Instrument (MNSI) as input features to classify patients into none, mild, moderate, and severe categories; the Extra Trees model demonstrated the best predictive performance (33). Stefanopoulos et al. (34) proposed a prediction model for amputation risk in diabetic foot ulcer patients. They used five variables: gangrene, peripheral vascular disease, weight loss, systemic infection, and osteomyelitis, and found that ensemble models and random forest algorithms provided the most accurate predictions (34).

### Internet of things applications research on diabetic foot

The Internet of Things (IoT) is a technological framework in which various devices such as sensors and wearable equipment are connected through the internet. This allows them to collect data, transmit information, and perform automated control. In studies on diabetic foot monitoring, many researchers have focused on integrating sensors with artificial intelligence to conduct analysis and improve clinical assessment. Agrawal et al. (35) utilized insoles embedded with continuous plantar pressure sensors for the detection and classification of diabetic foot conditions. They trained machine learning models for predictive analysis and demonstrated effective differentiation between healthy feet and diabetic feet (35). Shin et al. (36) applied machine learning methods to perform electrophysiological analyses among groups classified as normal, possible, and probable, among diagnosed diabetic mellitus (DM) patients. Their study achieved good identification results, particularly for diabetic sensorimotor polyneuropathy (DSPN) detection (36). Chen et al. (37) employed a wearable plantar pressure measurement device to capture foot pressure distributions during walking. Artificial intelligence models were then used to classify pressure images across different foot regions. Their study found that pressure readings from the first toe region and continuous walking for 10 min resulted in higher classification accuracy (37). Haque et al. (38) trained models using 19 years of data from the Epidemiology of Diabetes Interventions and Complications (EDIC) study, focusing on Michigan Neuropathy Screening Instrument (MNSI) features. The top 10 important features included Appearance of Feet (right), Ankle Reflexes (right), Vibration Perception (left and right), Appearance of Feet (left), 10-gm Filament Test (left and right), Bed Cover Touch, and Ulceration (right). The XGBoost model demonstrated the best predictive performance (38). Radunovic et al. (39) employed the Moveo device, which integrates machine learning algorithms to detect and monitor diabetic neuropathy. This device collects motion signals via four sensors placed on the hands and feet; it showed a strong correlation with traditional electrodiagnostic examinations (EDx) (39). Haque et al. (38) also applied an Adaptive Neuro-Fuzzy Inference System (ANFIS) to classify the severity of DSPN using electromyography (EMG) signals collected during walking from the vastus lateralis (VL), tibialis anterior (TA), and medial gastrocnemius (GM) muscles (40).

### Synthesis of results

**Figure 2:**
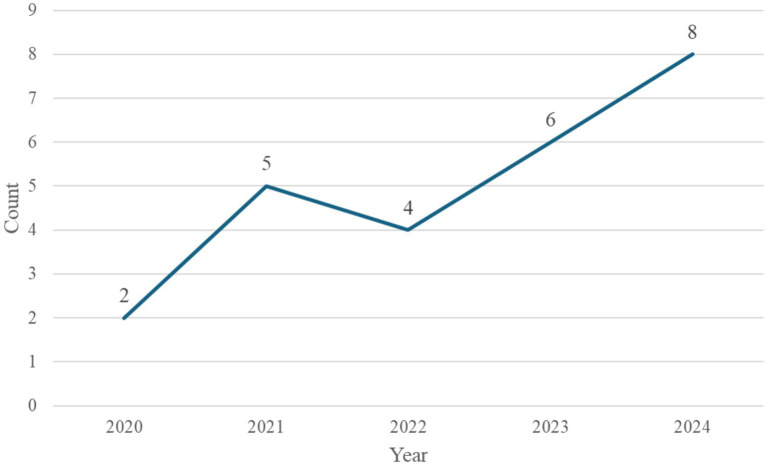
Annual publications from 2020 to 2024.

This study first categorized the collected literature based on the year of publication, with the annual distribution shown in Figure 2. The perceived trend indicates a steady increase in the number of publications applying machine learning to diabetic foot research, reflecting the growing academic interest in this field. The number of relevant articles rose from only two in 2020 to eight in 2024. This upward trend highlights the increasing recognition of the importance of diabetic foot issues and suggests that the application of artificial intelligence technologies in this domain holds strong potential for further development, making it a promising area for continued research.

**Figure 3:**
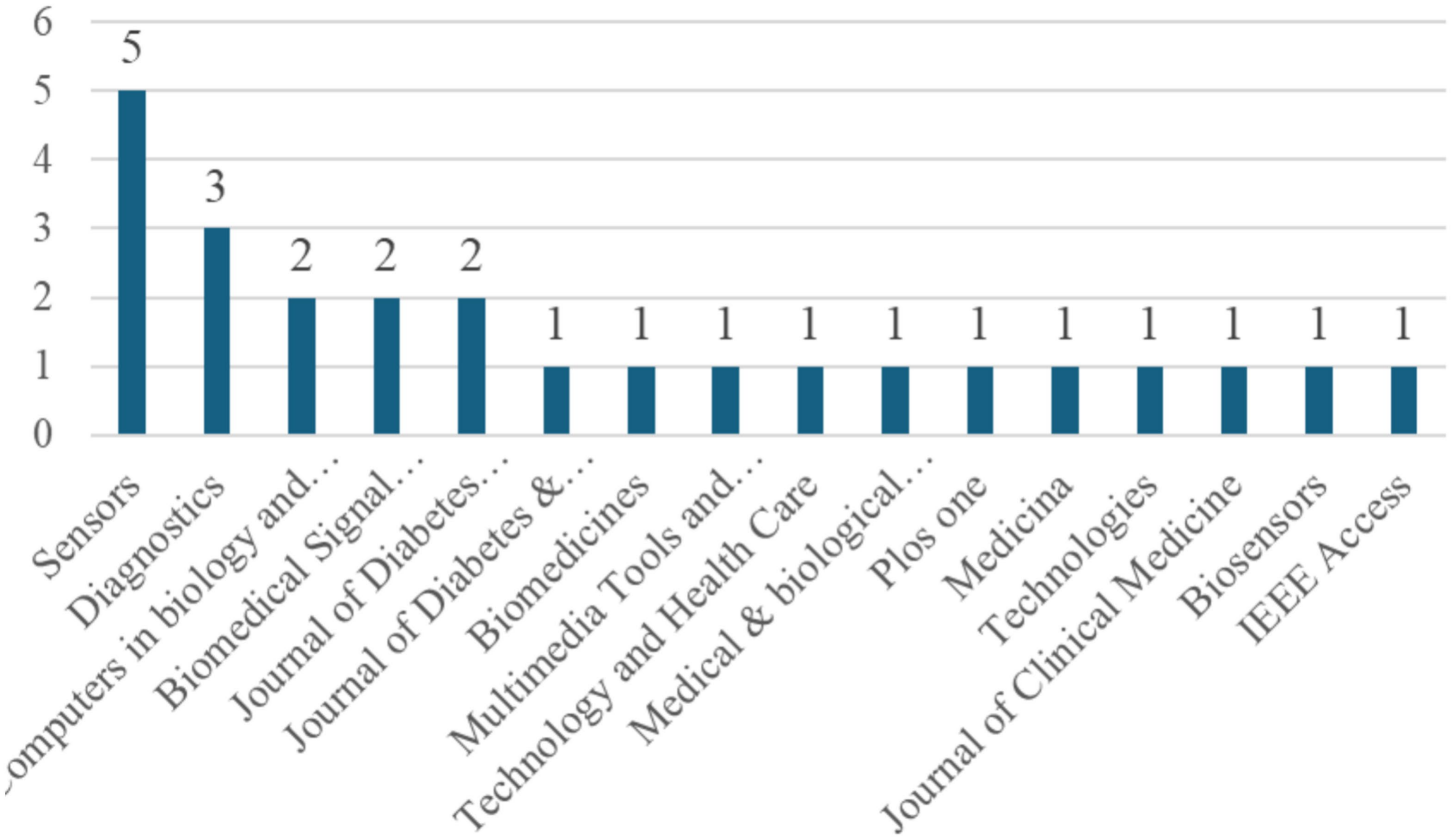
Distribution of journal publications.

This study further analyzed the distribution of journals in which the selected articles were published, as shown in Figure 3. The results indicate that Sensors had the highest number of publications, with a total of five articles, followed by Diagnostics, which published three articles. Computers in Biology and Medicine, Biomedical Signal Processing and Control, and the Journal of Diabetes Science and Technology each published two articles. Other journals contributed only one article each. These findings suggest that research on the application of machine learning in diabetic foot studies is primarily concentrated in a few specific journals, reflecting the growing recognition of this research topic within specialized academic outlets.

Finally, this study analyzed the average citation counts of articles published in each journal to assess the research impact of machine learning applications in the diabetic foot domain and to provide a reference for future scholars in selecting journals for submission and citation. Based on the ranking of average citation counts, the top five journals were: Multimedia Tools and Applications (190 citations), Computers in Biology and Medicine (62 citations), Sensors (47.4 citations), Medical & Biological Engineering & Computing (37 citations), and IEEE Access (20 citations). When considering only journals that published at least two articles, Computers in Biology and Medicine (62 citations) and Sensors (47.4 citations) demonstrated the highest average citation performance (Table 2).

**Table 2:** Statistical table of journal publication.

Rank	Journal	Documents	TC	D|TC
1	Multimedia Tools and Applications	1	190	190.0
2	Computers in Biology and Medicine	2	124	62.0
3	Sensors	5	237	47.4
4	Medical and Biological Engineering and Computing	1	37	37.0
5	IEEE Access	1	28	28.0
6	Plos one	1	20	20.0
7	Biomedical Signal Processing and Control	2	34	17.0
8	Journal of Clinical Medicine	1	17	17.0
9	Diagnostics	3	28	9.3
10	Technology and Health Care	1	8	8.0
11	Journal of Diabetes Science and Technology	2	15	7.5
12	Biomedicines	1	7	7.0
13	Biosensors	1	5	5.0
14	Journal of Diabetes and Metabolic Disorders	1	3	3.0
15	Medicina	1	2	2.0
16	Technologies	1	2	2

TC, total citations; D|TC, average number of citations per article.

## Discussion and conclusion

This study primarily utilized the Web of Science (WoS) database to conduct a literature analysis, focusing on journal articles published between 2020 and 2024. A total of 25 articles were included. Several key findings emerged from this review. There has been a consistent increase in the number of studies applying machine learning to diabetic foot research, indicating growing academic and clinical interest. Among these, image analysis was the most frequently addressed topic, with 12 studies identified. Within this group, thermal imaging was the dominant modality, featured in 10 studies, while general clinical imaging was addressed in only 2. Additionally, 7 articles analyzed structured clinical numerical data, and 6 focused on Internet of Things (IoT) applications. A common approach involved the use of sensor-integrated insoles for real-time monitoring of foot conditions, which suggests promising potential for translation into daily clinical practice.

This review focused on three main domains: image-based analysis, clinical data modeling, and IoT technologies. However, other emerging and relevant areas, such as genomics, proteomics, and metabolomics, were not included within the scope of this analysis. Future research should aim to integrate molecular and genetic information, which could enhance the predictive power of machine learning models and provide new insights into the underlying mechanisms of diabetic foot pathology. In terms of methodology, most of the included studies employed conventional machine learning techniques. More advanced approaches such as deep learning and large language models, which have demonstrated superior performance in handling complex and heterogeneous datasets, were underrepresented. Incorporating these techniques may significantly expand the analytical depth and improve predictive accuracy.

Most current studies have rarely performed external validation, raising concerns about model overfitting and limited generalizability to broader patient populations. Moreover, there is a lack of in-depth critical appraisal of the inherent limitations of existing machine learning models. Issues such as insufficient dataset diversity, lack of demographic representativeness, and potential algorithmic bias are often overlooked. These factors may lead to prediction errors and diminished performance when models are applied in real-world clinical settings.

In conclusion, while this review highlights encouraging developments in the application of machine learning to diabetic foot research, it also reveals critical gaps that must be addressed. Future studies should prioritize external validation, increase dataset diversity, incorporate bias mitigation strategies, and adopt emerging AI methodologies. Only through such efforts can the field move toward more reliable, equitable, and clinically useful machine learning applications for diabetic foot management.

## Data Availability

The original contributions presented in the study are included in the article/supplementary material, further inquiries can be directed to the corresponding authors.
